# Variations in Vaginal, Penile, and Oral Microbiota After Sexual Intercourse: A Case Report

**DOI:** 10.3389/fmed.2019.00178

**Published:** 2019-08-07

**Authors:** Miguel Carda-Diéguez, Nívia Cárdenas, Marina Aparicio, David Beltrán, Juan M. Rodríguez, Alex Mira

**Affiliations:** ^1^Department of Health and Genomics, Center for Advanced Research in Public Health, FISABIO, Valencia, Spain; ^2^Department of Nutrition and Food Science, Complutense University of Madrid, Madrid, Spain; ^3^Centro de Diagnóstico Médico, Ayuntamiento de Madrid, Madrid, Spain; ^4^Network of Epidemiology and Public Health, CIBERESP, Madrid, Spain

**Keywords:** microbiota, vagina, penile, oral, oral sex, bacterial vaginosis, *Lactobacillus*

## Abstract

**Background:** Bacterial vaginosis is the most common infection in women and it has been proved that dysbiosis of vaginal microbiota can promote the infectious status. This case report shows the effect of oral and vaginal sex over the microbiota of a heterosexual couple who reported repeated problems of vaginal and oral infections after sexual intercourse.

**Case Presentation:** A woman (32) reported to have vaginal infections and gingivitis after she had started a relationship with a man (34) and associated them with unprotected sex. No treatments successfully removed the problem and it repeated every time they had sexual encounters. Vaginal, penile and oral swabs were collected before and after sexual encounters in order to analyze changes in the respective microbiotas. DNA was extracted from all samples and the bacterial 16S rRNA gene was sequenced using Illumina MiSeq.

**Conclusions:** *Lactobacillus* occupied the great majority of the vaginal microbiota in all scenarios except after unprotected sex, which caused a bacterial dysbiosis that lasted at least for a week. Similarly, the penile microbiota changed significantly after unprotected sexual relationships. Interestingly, both oral and vaginal sex increased the abundance of *Lactobacillus* in the male oral and penile microbiota, respectively. In conclusion, unprotected sexual intercourse influenced the genital microbiota in the couple studied and future studies with larger sample sizes should study if sex may be a factor promoting vaginal infection through dysbiosis and hampered protection by the resident microbiota.

## Introduction

Bacterial vaginosis (BV) is the most common vaginal infection for women in reproductive period (1). It is estimated that 21 million adult women are affected by this condition every year in the United States (1). Studies of vaginal microbiota have associated several features to BV, including a reduction in hydrogen peroxide-producing *Lactobacillus*, a prevalence of anaerobic bacteria (*Prevotella, Gardnerella, Atopobium*, etc.), alkalinization, fishy odor and grayish-white vaginal discharge (2). Similar to the gut, commensal bacteria in vaginal microbiota have an essential role in maintaining the homeostasis and, consequently, a “healthy” status (3). Under health conditions, the vaginal microbiota has been proved to be mainly colonized by *Lactobacillus* (4) and some species have been used as commercial probiotics (5, 6). Dysbiosis of this microbiota has been related to reproductive disorders, such as pelvic inflammatory disease, endometriosis, ectopic pregnancy, preterm birth, and tubal factor infertility (7–10).

Interestingly, evidence of the direct role of an active sexual life in favor of a dysbiosis and the transmission of BV-associated microbiota has been reported in previous studies (11–13). Zozaya et al. described in 2016 the microbial communities in the penile skin, male urethra, and vagina with or without BV of monogamous heterosexual couples (14). They concluded that men had more similar microbiota to their female sexual partner who had BV than with other women who did not have intercourse with and suggested that sexual activity may promote the interchange of microbiota associated to BV.

## Methods

### Case Presentation

Here, we report the case of a woman [32 years old] with no previous episodes of vaginal and oral infections, who developed recurrent vaginal problems and gingivitis after a monogamous sexual relationship was started with a man [34 years old]. On the contrary, the male partner was previously diagnosed with chronic urethra infection and urethral stenosis and, as a consequence of later, he had been surgically treated before this relationship. Initially, the woman was empirically diagnosed as having candidiasis on the basis of the symptoms (vaginal discomfort) and unsuccessfully treated with cotrimazole (topical) and fluconazol (oral). After culturing a sample of vaginal exudate and, also, a sample of semen from her partner (see below), she was diagnosed as having BV and treated, again unsuccessfully with metronidazole and, later, with hydrogen peroxide (H_2_O_2_) intravaginal washes.

### Sample Collection and Bacterial Culture

Oral and genital swabs were collected by the patients and the composition of oral, vagina, and penile microbiotas were analyzed by bacterial culture and by Illumina sequencing of the V3-V4 region of the 16S rRNA gene before and after sex. Oral samples were collected with a sterile swab by brushing along the inside of the subject's cheek for 10 s. Vaginal samples were obtained by inserting a sterile swab through the vaginal introitus to ~5 cm; and were then rotated twice. Preputial cultures were taken after the foreskin was retracted carefully and, then, the culture swab was swept circumferentially once around the surface of the glans starting proximally to the urethral meatus. During an initial visit to the gynecologist both members of the couple were instructed how to collect their respective samples in order to be able to self-obtain the specimens analyzed in this study.

In addition, the effects of H_2_O_2_ over the vaginal and penile microbiota were assessed after 1 and 15 days of treatment. In accordance with the Declaration of Helsinki, all volunteers gave written informed consent to the protocol, which had been approved (protocol 29/017-E) by the Ethical Committee of Clinical Research of Hospital Clínico San Carlos Madrid (Spain).

Samples were diluted in peptone water and spread onto Columbia Nalidixic Acid (CNA), Mac Conkey (MCK), Sabouraud Dextrose Chloramphenicol (SDC), and Gardnerella (GAR) agar plates (BioMerieux, Marcy l'Etoile, France). They were also spread onto agar plates of MRS (Oxoid, Basingstoke, UK) supplemented with either L-cysteine (0.5 g/L) (MRS-C) for isolation of lactobacilli. All the plates were incubated for 48 h at 37°C in aerobic conditions, with the exception of the MRS-C and MRS-B ones, which were incubated anaerobically (85% nitrogen, 10% hydrogen, 5% carbon dioxide) in an anaerobic workstation (DW Scientific, Shipley, UK). Identification of the bacterial strains (at least one isolate of each colony morphology per medium and per sample) was performed by MALDI-TOF (VITEK MS, BioMerieux).

### DNA Extraction, Amplification, and Sequencing

DNA was extracted using the MagNa Pure LC DNA Isolation kit II (Roche^®^) and a MagNa Pure Instrument. Protocol was used as indicated by the company with some modifications following (15).

Following Dzidic et al. (15), DNA was cleaned, measured by fluorimetry and the V3-V4 hypervariable region of the 16S rRNA gene was amplified using universal primers optimized for Illumina sequencing (15). Library was constructed following the 16S rRNA gene Metagenomic Sequencing Library Preparation Illumina protocol (Part #15044223 Rev. A) and sequenced at the sequencing service at the FISABIO Institute (Valencia) using 2 × 300 bp paired-end Illumina protocol.

### Data Processing

Dada2 was used to filter, trim, denoise, and mergepairs reads (16). First of all, reads were filtered for adapters and primers and then, a 50 bp minimum length, quality of 35, none Ns, and singletons reads filters were applied. The remaining reads were merged, clustered and cleaned for host and chimeric reads and, finally, assigned to a taxon using SILVA non-redundant database (17). Principal component analyses (PCoA) was performed using R.

## Results

Two samples failed in the sequencing procedure and the number of reads was too small to proceed with the analyses (Supplementary Table 1). All other samples had a mean number of 51,000 sequences after eliminating PCR chimeras and filtering reads by quality and length (15).

### Bacterial Cultures

A series of vaginal and penile samples of the patients were cultured in order to study which cultivable bacteria were present and in which quantities. *Gardnerella vaginalis* and *Candida albicans* were only detected in the vagina while *Dermobacter hominis, Corynebacterium jeikeium*, and *Staphylococcus haemolyticus* were only isolated in the penile. *Finegoldia magna* was one of the most abundant species in both the penile and vagina (4.5 × 10^6^ cfu/ml) (Table 1). Other species were detected in similar levels to *Finegoldia* in the vagina (*Gardnerella vaginalis* and *Corynebacterium amynocolatum)* and in the penile (*Enterococcus* sp.).

**Table 1:** Culture-based analysis of a sample of vaginal exudate (cfu/swab) and a sample of semen (cfu/ml) provided by the couple.

**Species**	**Man**	**Woman**
*Candida albicans*	ND	2.0E+04
*Corynebacterium amycolatum/xerosis*	ND	4.5E+06
*Corynebacterium jeikeium*	1.5E+04	ND
*Corynebacterium tuberculostearicum*	3.5E+02	ND
*Dermobacter hominis*	2.1E+04	ND
*Escherichia coli*	2.5E+02	1.4E+02
*Enterococcus* spp.	2.5E+06	2.5E+02
*Finegoldia magna*	4.5E+06	4.5E+06
*Gardnerella vaginalis*	ND	1.0E+06
*Klebsiella pneumoniae*	2.0E+02	4.0E+04
*Staphylococcus aureus*	2.5E+04	1.5E+03
*Staphylococcus epidermidis*	1.0E+05	2.0E+05
*Staphylococcus haemolyticus*	1.0E+05	ND
*Staphylococcus lugdinensis*	2.0E+05	2.3E+02
*Streptococcus mitis/oralis*	7.5E+02	1.5E+04

*ND, not detected*.

### 16S rRNA Microbiota Composition

**Figure 1:**
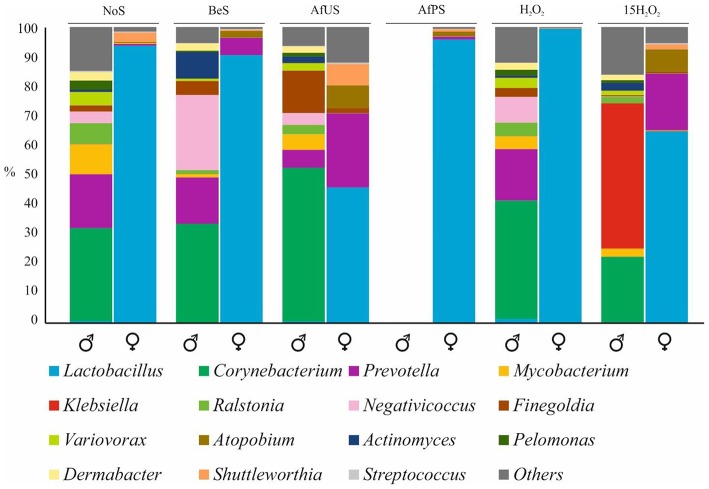
Microbial composition of penile and vaginal swabs. Most abundant genus were plotted in different colors. NoS, microbiota without sexual intercourse; BeS, immediately before sexual encounter; AfUS, 4 days after unprotected sexual encounter, no oral sex and with ejaculation inside the vagina; AfPS, immediately after vaginal sex with condom and oral sex; H_2_O_2_ after 1 day of H_2_O_2_ treatment; 15H_2_O_2_, after 15 days of intermittent H_2_O_2_ treatment. Male and female samples are indicated with the corresponding symbol.

Vagina and penile control samples were obtained 4 days before sex. Since no symptoms of BV were present, we considered it as the “healthy” or “NoS” (No sex). Bioinformatic analyses showed that normal microbiota in the penile was mainly colonized by *Corynebacteruim* (31%)*, Prevotella* (18%)*, Mycobacterium* (10%)*, Ralstonia* (7%), and *Negativicoccus* (3.9%) (Figure 1). In the vagina, *Lactobacillus* reached over 90% of the bacterial community (Figure 1). In particular, sequence similarity analyses showed that *L. iners* was the dominant species in the vagina. Remarkably, the above-mentioned most abundant species by sequencing were in very low concentration in the cultures, and *L. iners* was not detected.

### Effect of Vaginal Sex on Genitals Microbiota

With the aim of studying the consequences of sex encounters in the vaginal- and penile-associated microbiotas of the couple under study, both genitals were sampled immediately before sex (samples coded as BeS) and 4 days after unprotected sex (AfUS). Moreover, in order to identify any microbial changes not caused by direct genital contact, vaginal and penile samples were also taken immediately after protected (condom) sex (AfPS).

Having sampled twice vagina and penile before any sex activity (NoS and BeS) allowed us to assessed the stability of these microbiotas over time. The data show that both microbiotas remained stable and only minor changes were detected (Figure 1). *Corynebacterium* and *Lactobacillus*, the major genera in both cases, prevail as the most abundant genera and their abundances did not vary significantly. Thus, after-sex samples were compared to BeS.

**Figure 2:**
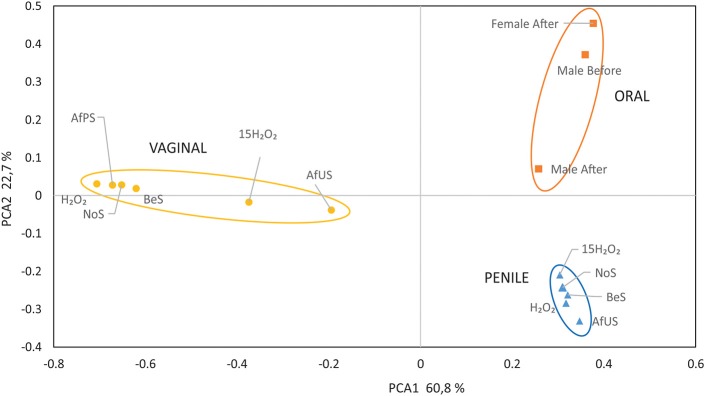
Principal component analyses of the associated microbiota to the penile, vaginal, and oral samples. NoS, microbiota without sexual intercourse; BeS, immediately before sexual encounter; AfUS, 4 days after unprotected sexual encounter, no oral sex and with ejaculation inside the vagina; AfPS, immediately after vaginal sex with condom and oral sex; H_2_O_2_ after 1 day of H_2_O_2_ treatment; 15H_2_O_2_, after 15 days of intermittent H_2_O_2_ treatment.

When BeS and AfUS microbiotas were compared we found remarkable changes in both vagina and penile samples. Interestingly, the percentage of *Lactobacillus* was reduced considerably (from 90 to 45%) in the vagina after having sexual relationships without protection while in the penile, the proportions of *Corynebacterium* increased from 33 to 52% (Figure 1). Moreover, *Prevotella* and *Atopobium*, usual BV-associated bacteria, increased in the vagina after sexual intercourse. Considering that both genitals were sampled 4 days after sex, the variations found suggest that the consequences of unprotected sex for the microbiota are major and last at least for several days, especially in the vagina. On the contrary, no significant changes were detected in the vagina when BeS and AfPS were compared, and *Lactobacillus* remained as the main genus in the vagina. In addition, when all samples were plotted in a PCoA, the distribution of the samples regarding the vaginal microbiota showed higher proximity between the microbiota BeS and AfPS than with AfUS (Figure 2). This would mean that microbial changes appeared only when penile contact is made and those could be avoided with condoms. Unfortunately, the penile sample AfPS did not amplify properly and we could not get enough reads to proceed with the taxonomic analyses.

**Figure 3:**
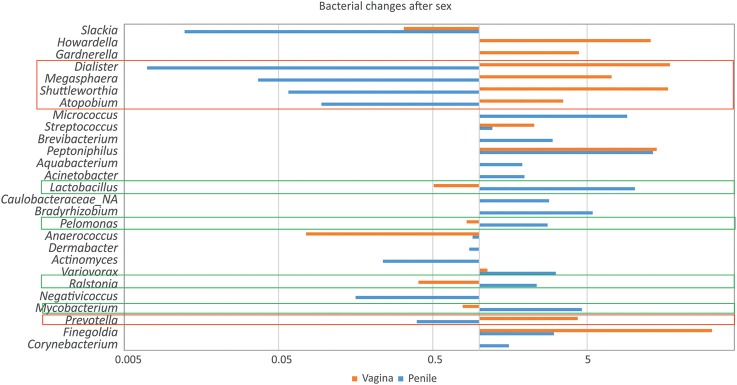
Microbial variations in genital swabs associated to sexual encounters. The fold change of genera was calculated in vaginal and penile samples before and after sexual encounter. The log of this fold change was plotted for those which had the biggest variations. Genera which proportions increased in the vagina but reduced in the penile, or vice versa, were highlighted in red and green boxes, respectively.

In order to observe the most significant taxonomical changes, the AfUS/BeS ratios of those genera with more than 1% abundance in the vagina or penile were calculated (Figure 3). The variations observed show some genera that increased its abundance in the vagina and reduced its levels in the penile, or vice versa. On one hand, *Lactobacillus, Pelomonas, Ralstonia*, and *Mycobacterium* increased in the penile from 2 to 10 times (Figure 3) while decreased in the vagina (x1.2–2.4). On the other hand, *Dialister, Megasphoera, Shuttleworthia, Atopobium*, and *Prevotella* decreased in the penile (x2.5–142) and augmented in the vagina (x3.5–17). This suggests that a transmission of some members of the genitals microbiota may be taking place when those are in contact, in agreement with previous studies as Vodstrcil et al. who detected a transmission of commensals and potential pathogenic clades during vaginal-sex (18).

### Effect of H_2_O_2_ in Vaginal and Penile Microbiota

Since no medical treatment appeared to alleviate the symptoms of the sex-derived pathology, the couple was prescribed to perform H_2_O_2_ washes (penile washes and intravaginal irrigations). We analyzed the microbiota after one (H_2_O_2_) and 15 days (15H_2_O_2_) of treatment. In both genitals, microbiota composition was not affected after 1 day of treatment and the sample clustered with the rest of samples from its corresponding niche (Figure 1). On the contrary, several genera were significantly affected after 15 days of intermittent washing. For example, *Lactobacillus* maintained its high proportions in vagina (99.3%) after 24 h, but after 15 days of H_2_O_2_ treatment, the vaginal profile was more similar to the microbiota AfUS, since *Lactobacillus* reduced its proportions to 64.7% and *Prevotella* and *Atopobium* increased to 19.11 and 7.5%, respectively. On contrary, *Prevotella* was eliminated in the penile after 15 days of this treatment.

As mentioned before, *Prevotella* and *Atopobium* are not desired members in a healthy vaginal microbiome. Consequently, their increase, putatively caused by the H_2_O_2_ treatment, would be the opposite to that expected to solve the BV. Furthermore, 15 H_2_O_2_ samples clustered closer to AfUS than the rest of vaginal samples in the PCoA (Figure 2). Interestingly, the most significant change in penile microbiota caused by H_2_O_2_ was a remarkable increase in *Klebsiella* (49%). Meanwhile, major components of the penile microbiota such as *Mycobacterium, Finegoldia* and *Ralstonia* decreased after the treatment.

The results obtained after sequencing the genital microbiotas treated with H_2_O_2_ suggested that, in both cases, a prolonged treatment is necessary to significantly change the microbial population although the changes appeared to be opposite to those required to solve the BV. Moreover, the remarkable increase of *Klebsiella* indicated a potentially high resistance of this genus to H_2_O_2_ in the penile.

### Effect of Oral Sex in Oral Microbiota

Gingival problems present in the female patient were recurrent to sexual activity. Thus, we studied the oral microbiota before and after oral sex to study its variations in both patients and to detect putative gingival-associated pathogens. Unfortunately, the oral sample of the female before oral sex did not amplify at sufficient levels and the number of reads obtained was too small to consider the sample for analyses (Supplementary Table 1). We therefore compared male oral microbiotas before and after oral sex and found that the oral microbiota changed. The most significant variations included *Streptococcus*, which reduced its abundance from 49,8 to 14% and *Lactobacillus*, which increased its abundance 63 times to reach 2,8% (Supplementary Figure 1). Regarding the rest of genera abundant in this sample, only *Prevotella* increased considerably (3.5 times).

## Discussion

We reported a case of a patient with bacterial vaginosis and gingivitis caused by sexual intercourse. Even though sequencing the 16S rRNA can have some biases associated to the amplification steps (19), it is generally accepted that the 16S sequencing results are accurate to reality and also that cultivable bacteria are a small percentage of the whole microbiota in almost every niche (20). Thus, we are considering 16S rRNA sequencing data as the main information source to study bacterial composition shifts in this case report.

In order to analyze the putative changes that sex produced in the genitals and oral cavity of the patients, we sampled vaginal, penile, and oral swabs before and after sexual intercourse. Significant alterations in the microbial composition were detected, especially in the vagina, which might explain the tendency to develop BV after sexual intercourse. For example, the decrease in *Lactobacillus* would be understood as a detrimental variation in the vagina since the protective role of members of *Lactobacillus* is well-known. In addition, *L. iners*, the dominant species in the patient's vagina, has been determined as the most abundant *Lactobacillus* species in women with lower concentrations of D-lactic and with higher risk of BV (4, 21, 22). *L. iners* is a heme-negative species which requires blood supplementation to grow in MRS. This could explain why it was not isolated in this case.

Moreover, *Prevotella* and *Atopobium*, two genera commonly found in vaginal microbiota and related with vaginal dysbiosis and also negatively related with the abundance of *Lactobacillus* (23–25), increased its proportions in the vagina after contact with the penile. However, the presence of both genera in the vagina when a condom was used was minor. We conclude that the origin of these organisms could be the penile and therefore, that there probably was a direct transmission of potential vaginal pathogens through vaginal-sex.

The few available studies in which the penile microbiome has been studied appear to agree with our data. For example, *Corynebacterium* had already been described as the main genus in the penile (14, 26). When sexual intercourse occurred, we found an increase of the *Lactobacillus* proportions in the penile after unprotected vaginal sex. Because of the low abundance of this genus in the penile when no sex occurred and its predominance in the vagina, we think that a vaginal origin of this genus is likely. This supports, the idea of microbiota transmission from one partner to another through vaginal sex. It is also remarkable that microbiota changes persisted after 4 days. This suggests that sexual activities not only would change the genitals microbiota but also could last for several days, allowing putative pathogens to colonize and/or overgrow. Moreover, when sex was practiced with condom this transmission was not detected, supporting the idea that a direct contact with the penile and the vagina is needed to transmit or favor the growth of these microorganisms.

In addition to the vagina, the female patient also complained about gingival pain. The study of the oral microbiota before and after having oral sex showed a significant reduction in the genus *Streptococcus* or an increase in *Lactobacillus*, both in the male mouth. Similarly to the penile, we hypothesized that the increase in *Lactobacillus* could be caused by vaginal contact. As far as we know, this is the first report of bacterial transmission through oral sex and the first to study these variations, and therefore more data are needed to establish bacterial shifts in oral microbiota after oral sex. It must be kept in consideration that microbial transference could also occurred through kissing (saliva) before and after oral sex.

Finally, sequencing results indicated that H_2_O_2_ treatment had no effect on microbiota composition after 24 h treatment but both penile and vagina microbiota were affected significantly after 15 days. However, the observed changes did not suggest an improvement, especially in the vagina, where H_2_O_2_ treatment reduced the proportions of *Lactobacillus* and increased *Prevotella* after 15 days. The fact that Prevotella decreases in the penile but augmented in the vagina with the same treatment is probably caused by the environmental conditions in the respective niches (pH, humidity, etc.). *Klebsiella* appeared to be the only genus which increased considerably in the penile after 15 days of treatment, which is consistent with this genus being able to produce catalases (27).

In conclusion, the data indicate that unprotected sex in the reported couple induced a bacterial dysbiosis in the genitals and suggest that aggressive antibacterial treatments alone may not be effective in restoring homeostasis. It has yet to be determined if vaginal changes are caused by microbial transmission or by antimicrobial components produced by penile-associated bacteria. Future work in more individuals should also establish whether this effect of unprotected sexual intercourse on bacterial dysbiosis can be extended to the general population or what are the factors increasing its risk.

## Data Availability

The datasets generated and/or analyzed during the current study are available in the SRA repository, with accession number PRJNA524629.

## Ethics Statement

In accordance with the Declaration of Helsinki, all volunteers gave written informed consent to the protocol, which had been approved (protocol 29/017-E) by the Ethical Committee of Clinical Research of Hospital Clínico San Carlos Madrid (Spain).

## Author Contributions

NC, MA, and DB were responsible for the bacterial cultures and sampling. JR and AM designed the study. MC-D performed the bioinformatic analyses and wrote the paper. JR and AM revised the manuscript. The results were analyzed and conclusions were made with the participation of JR, AM, and MC-D.

### Conflict of Interest Statement

The authors declare that the research was conducted in the absence of any commercial or financial relationships that could be construed as a potential conflict of interest.

## Abbreviations

NoS: microbiota without sexual intercourse
BeS: immediately before sexual encounter
AfUS: 4 days after unprotected sexual encounter, only penetration and with ejaculation inside the vagina
AfPS: immediately after vaginal sex with condom and oral sex
H2O2: after 1 day of H2O2 treatment
15H2O2: after 15 days of intermittent H2O2 treatment
PCoA: principal component analyses.
