# Corrigendum: Variations in Vaginal, Penile, and Oral Microbiota After Sexual Intercourse: A Case Report

**DOI:** 10.3389/fmed.2019.00294

**Published:** 2020-01-09

**Authors:** Miguel Carda-Diéguez, Nívia Cárdenas, Marina Aparicio, David Beltrán, Juan M. Rodríguez, Alex Mira

**Affiliations:** ^1^Department of Health and Genomics, Center for Advanced Research in Public Health, FISABIO, Valencia, Spain; ^2^Department of Nutrition and Food Science, Complutense University of Madrid, Madrid, Spain; ^3^Centro de Diagnóstico Médico, Ayuntamiento de Madrid, Madrid, Spain; ^4^Network of Epidemiology and Public Health, CIBERESP, Madrid, Spain

**Keywords:** microbiota, vagina, penile, oral, oral sex, bacterial vaginosis, *Lactobacillus*

In the original article, there was a mistake in Figure 1 as published. The penile swab compositions for H_2_O_2_ and 15H_2_O_2_ were mistakenly swapped. Similarly, the vaginal swab compositions for H_2_O_2_ and 15H_2_O_2_ were also mistakenly swapped. The corrected Figure 1 appears below.

**Figure 1:**
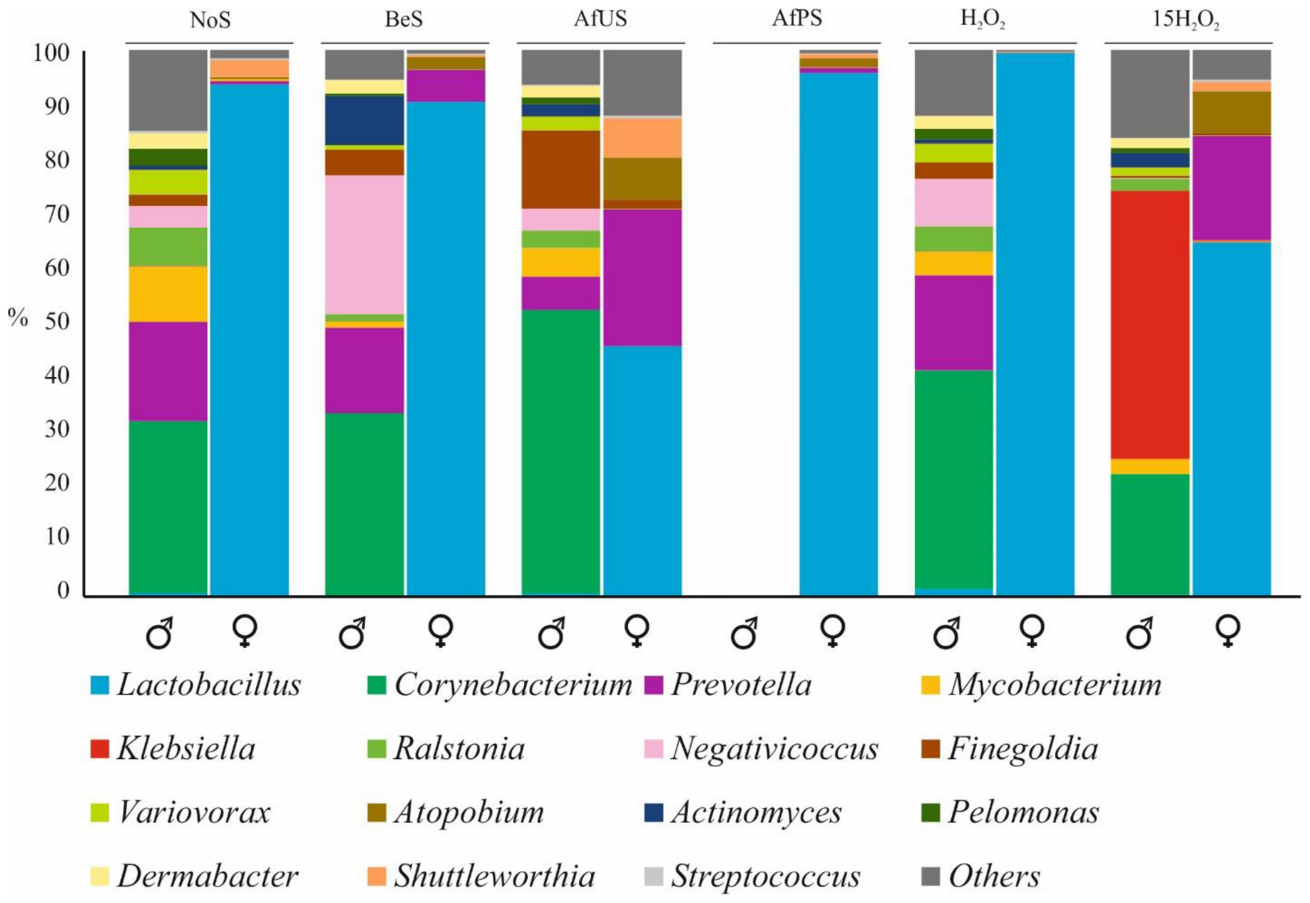
Microbial composition of penile and vaginal swabs. Most abundant genus were plotted in different colors. NoS, microbiota without sexual intercourse; BeS, immediately before sexual encounter; AfUS, 4 days after unprotected sexual encounter, no oral sex and with ejaculation inside the vagina; AfPS, immediately after vaginal sex with condom and oral sex; H_2_O_2_ after 1 day of H_2_O_2_ treatment; 15H_2_O_2_, after 15 days of intermittent H_2_O_2_ treatment. Male and female samples are indicated with the corresponding symbol.

The authors apologize for this error and state that this does not change the scientific conclusions of the article in any way. The original article has been updated.

