# Supplementary material for: Variations in Vaginal, Penile, and Oral Microbiota After Sexual Intercourse: A Case Report
Source: Front Med (Lausanne). 2019 Aug 7;6:178. doi: 10.3389/fmed.2019.00178 (PMC6692966; doi:10.3389/fmed.2019.00178)
Supplement: Supplementary file 1 [file Presentation_1.PPTX]

## Slide 1
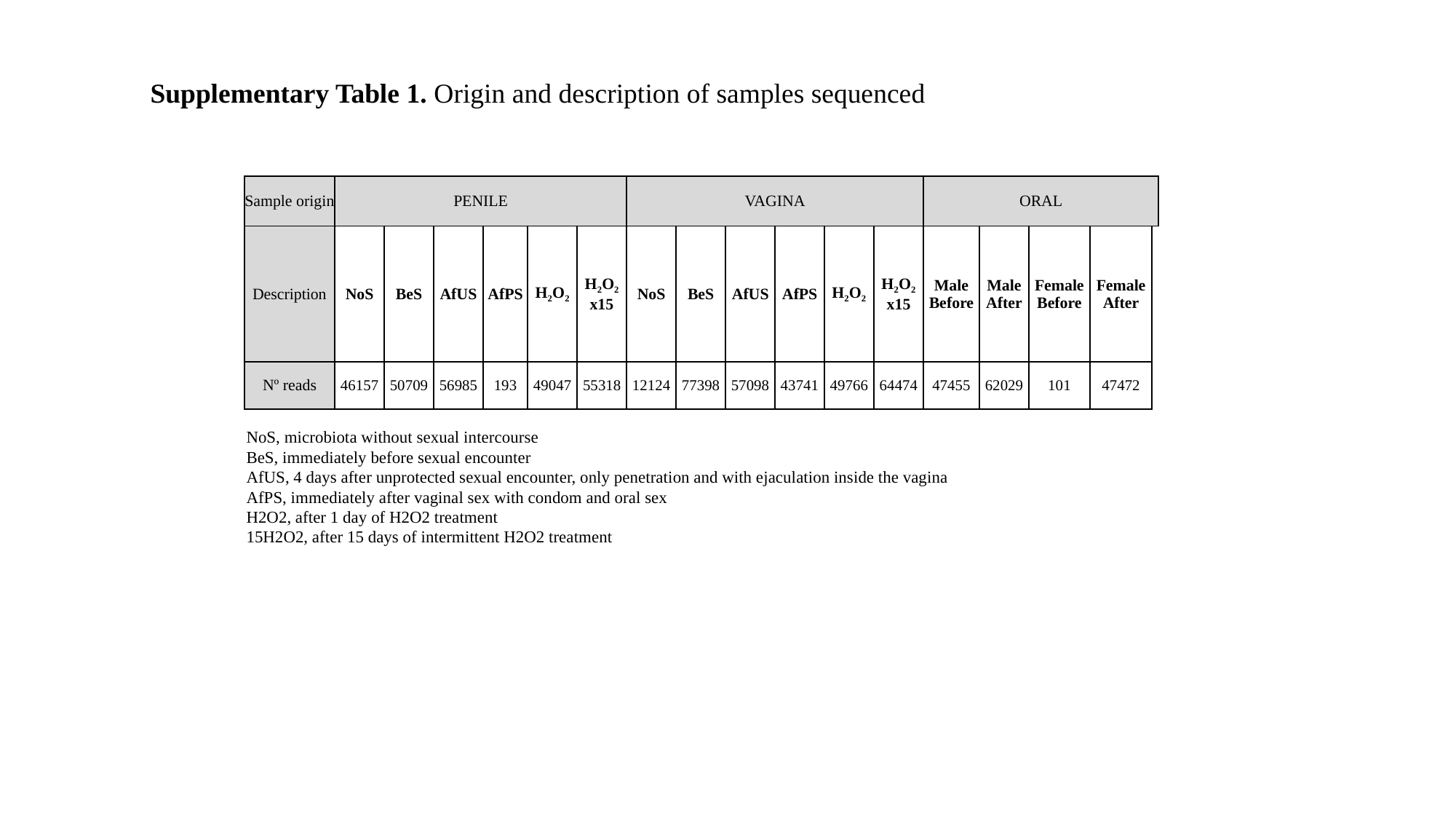

Supplementary Table 1. Origin and description of samples sequenced
| Sample origin | PENILE | | | | | | VAGINA | | | | | | ORAL | | | | |
| --- | --- | --- | --- | --- | --- | --- | --- | --- | --- | --- | --- | --- | --- | --- | --- | --- | --- |
| Description | NoS | BeS | AfUS | AfPS | H2O2 | H2O2 x15 | NoS | BeS | AfUS | AfPS | H2O2 | H2O2 x15 | Male Before | Male After | Female Before | Female After | |
| Nº reads | 46157 | 50709 | 56985 | 193 | 49047 | 55318 | 12124 | 77398 | 57098 | 43741 | 49766 | 64474 | 47455 | 62029 | 101 | 47472 | |
NoS, microbiota without sexual intercourse
BeS, immediately before sexual encounter
AfUS, 4 days after unprotected sexual encounter, only penetration and with ejaculation inside the vagina
AfPS, immediately after vaginal sex with condom and oral sex
H2O2, after 1 day of H2O2 treatment
15H2O2, after 15 days of intermittent H2O2 treatment

## Slide 2
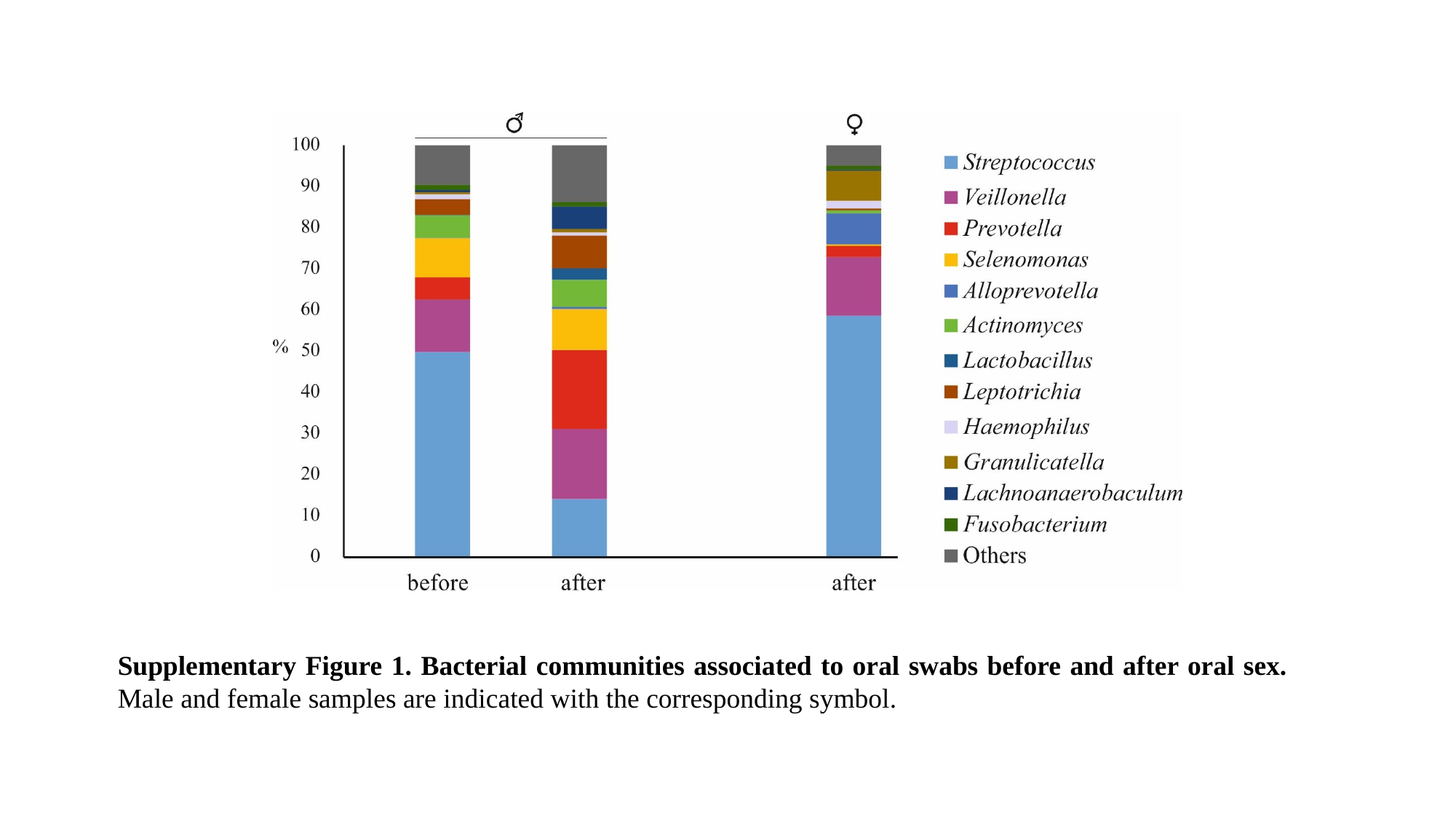

Supplementary Figure 1. Bacterial communities associated to oral swabs before and after oral sex. Male and female samples are indicated with the corresponding symbol.
